# Supplementary material for: Trust development as an expectancy-learning process: Testing contingency effects
Source: PLoS One. 2019 Dec 11;14(12):e0225934. doi: 10.1371/journal.pone.0225934 (PMC6905544; doi:10.1371/journal.pone.0225934)
Supplement: Attachment — Submitted filename: Responses to reviewers.docx [file pone.0225934.s004.docx]

**Responses to the Editor**

**1.  When submitting your revision, we need you to address these additional requirements.**

**Please ensure that your manuscript meets PLOS ONE's style requirements, including those for file naming.**

*We have completely reworked the document to comply with PLOS One's style requirements.*

**2. We note that you have indicated that data from this study are available upon request. PLOS only allowsdata to be available upon request if there are legal or ethical restrictions on sharing data publicly.**

*We have prepared the files and uploaded them as Supporting Information Files.*

**3. We note that on page 8 it is stated that the screen thanked children for feeding the donkey, yet participants in this part of the study were adults. Please correct and clarify.
Please note that according to our submission guidelines outmoded terms and potentially stigmatizing labels should be changed to more current, acceptable terminology. For example: “Caucasian” should be changed to “white” or “of [Western] European descent” (as appropriate).**

*This phrasing was altered as requested.*

**4. Please provide additional details regarding participant consent. Please ensure that the description of each of the experiments performed includes a description of the consent procedures. In the Methods section(s), please ensure that you have specified (1) whether consent was informed and (2) what type you obtained (for instance, written or verbal). Please state whether you obtained consent from parents or guardians for all studies including minors.**

*In study 1, adults' written consent was obtained. For both Studies 2 & 3, parent and child written consent was obtained.*

5. Please amend either the title on the online submission form (via Edit Submission) or the title in the manuscript so that they are identical.

XXX

**6. Please include a title for tables 1-3.**

*All tables received titles*

**Responses to the Reviewers**

**Response to Reviewer #1:**

**Overall this is an excellent set of studies that build on each other and present an important methodological approach to applying learning theory to trust development. The data are convincing and the methods are sound. I have a few requests:**

*We are thankful for this positive evaluation of our paper and grateful for the helpful suggestions thanks to which the paper was substantially strengthened.*

**1) The authors should probably refrain from referring to "marginally significant" results as this is technically a violation of frequentist statistical approaches from most points of view, although this is an admittedly minor issue.**

*We agree that it is wiser to be more cautious about valuing marginally significant effects. We thought it was most prudent to remove any discussion of marginally significant effects. Moreover, we no longer refer to marginally significant effects. Instead, where appropriate or necessary for the flow of the story we now more carefully describe that effects did not reach significance.*

**2)More importantly, it would be useful for the authors to provide both confidence intervals on point estimates and effect size information. This type of information is critical for determining the soundness of the results and understanding the degree of uncertainty with which the effects are estimated.**

*Following this request, we gave effect size information wherever possible. Moreover, we additionally provided confidence intervals for point estimates. Given the amount of analyses we could discuss, we wanted to keep the results section concise, so for now we decided to provide all the information for all the significant effects and for the non-significant effects that were important to make a theoretical point.*

**3) The results are in line with previous findings and theory, but offer important advances in methodology and a first guess as to what "responsiveness" or "good enough" really means. This type of work is critical for advancing the field's knowledge regarding attachment learning and trust development. Overall this manuscript is very well written and provides compelling evidence for the theoretical ideas therein.**

*We are very grateful for these words of praise!*

**4) A final note, similar work in the adult attachment literature has found similar evidence for the same learning theory based approach. The authors may be interested in these articles given the similarity in theory and approach.

Beckes, L. Simons, K., Lewis, D., Le, A., & Edwards, W. L. (2017). Desperately seeking support: Negative reinforcement schedules in the formation of adult attachment associations. Social Psychological and Personality Science, 8, 229-238.

Beckes, L., & Coan, J. A. (2015). The distress-relief dynamic in attachment bonds. In C. Hazan & V. Zayas (Eds.), Bases of Adult Attachment: Linking Brain, Mind, and Behavior (pp. 11-33). New York, NY, US: Springer Science + Business Media.

Beckes, L., Coan, J. A., & Morris, J. P. (2013). Implicit conditioning of faces via the social regulation of emotion: ERP evidence of early attentional biases for security conditioned faces. Psychophysiology, 50, 734-742.

Beckes, L., Simpson, J. A, & Erickson, A. B. (2010). Of snakes and succor: Learning secure attachment associations with novel faces via negative stimulus pairings. Psychological Science, 21, 721 – 728.**

*We are very grateful for this suggestion. We do know the work of Lane Beckes and highly appreciate his papers. Because we agree that mentioning this work strenghtenes the paper, we followed this suggestion. We discussed his work at two places in the paper.*

*First, in the introduction, we were asked by reviewer 2 (comments 1 & 2) to contrast our learning theory model of attachment development with attachment theory's traditional criticism on a learning theoretical approach to attachment. In the flow of this argumentation in the introduction, we now mention these papers to make the point that support has been found for such mechanisms in adult attachment research:*

p4: More recently, researchers started to argue that this biological preparedness of infants to establish attachment relationships with caregivers does not fully explain why individual differences in children's expectations or trust about caregivers' availability for support develop [8]. One thusfar understudied possibility could be that learning models might be a helpful addition to attachment theory to explain these differences [19]. Recent literature argues in a similar way that adult attachment development reflects a conditioning process [20-22].

*Second, in the discussion, we added critique on our paradigm, as we still lack convincing evidence that trust towards the avatar is attachment relevant. Although the Beckes et al., 2017 study did not directly target trust, the theoretical assumption and operationalization is very comparable and the results are also convergent with our studies. So we mentioned the latter paper to strengthen our argument that in spite of the validity of these concerns, our paradigm could still yield useful results.*

p27: This all suggests that more research and similar research with more ecologically valid designs is crucial in the future, In spite of these concerns, one experiment in adults has shown that manipulating the likelihood that an unknown supporter helps avoiding exposure to shocks affects participants' attentional processing and attachment-related appraisals of the supporter [36]. Although effects on trust were not measured in this study, it seems reasonable to assume that the manipulation could have affected trust as well.

**Responses to Reviewer #2:**

**The authors use a learning theory approach, seeking to illuminate the processes by which child-caregiver trust and help seeking develop. Using a task in which adults and children learn reward schedules from a "caregiver" the authors measured trust as a function of caregivers who are likely to provide the correct answer at different rates. The study is an interesting possible analogue to these processes and the learning theory account is welcome. I agree with the authors that such a framework is likely to yield specific hypotheses. At the same time, I have feedback the authors may consider:**

*Thank you for your generous feedback and appreciation of the paper. Using the feedback while revising the paper has significantly improved the manuscript.*

**1. One concern I have with the paper is that the authors say they are seeking to illuminate the "underlying developmental mechanism" of trust and help seeking with caregivers. However, their study can speak very little to these processes. It is unclear whether the results are consistent with reward learning in close relationships, or, alternatively, the authors would have found the exact same results if participants were told they were playing with a computer. The authors seek to capture a process, but only show that trust increases with certain rates of reward and not others, and help seeking is higher in some conditions than others. It is not clear, however, whether what they describe is consistent with the processes of developing trust or seeking help in attachment contexts. Just as much as these data could describe a process somewhat similar to developing trust in close relationships, it could just as easily describe adults and children learning reward rates in a laboratory task.**

*We agree that the current design, in spite of its interesting characteristics cannot rule out this alternative interpretation. Therefore, we devoted substantial attention to this issue in the limitations section. We now wrote:*

p29: More specifically, at this point one could argue that we merely measured learning rates of reward instead of processes related to attachment development. In line with this concern, we cannot rule out the possibility that similar effects could have been found had we instructed the children they were playing with a computer. Thus, it would be premature to argue that the current study contradicts attachment theory's traditional view that attachment is an inborn mechanism that does not require learning processes [18]. A next step would be to investigate whehter these findings translate to more naturalistic research. With that regard, it is promising that the current pattern of findings does not contradict existing more broadband attachment research.

**2. Similarly, it is possible that learning rates of reward explains these developmental processes, but this ignores the extensive evidence of an attachment system that predisposes children to maintain proximity and use caregivers as a source soothing. I wonder how the authors might reconcile findings from attachment theory with their own approach?**

*We agree that this is an important issue that we did not explicitly address in the previous version of the paper. In fact, this refers back to a very old discussion between the more evolutionary view on attachment as proposed by Bowlby and Ainsworth and learning theory like, for example, proposed by for example Gerwitz. This discussion has been settled in a seminal paper from Rajecki, Lamb, and Obmascher (1978). Ever since, it has been accepted that attachment does not develop due to learning experiences.*

*However, historically, Bowlby has never been fully opposed against the idea that learning processes could partly explain aspects of attachment development. He did not touch this issue in his published work, but he referred to this in his correspondence with colleagues. Thanks to Robbie Duschinsky, we got access to some of these letters and we mentioned them in the new version of the paper.*

*Moreover, it should be noted that the argumentation of Rajecki et al. more refers to why children develop an attachment bond to parents and less to why individual differences in attachment (in)security develop. Recent work increasingly argues that with regard to that issue, learning theory could prove to be an added value. We now describe this debate and this argumentation in the introduction.*

p4:

The idea that learning theory could explain at least a part of attachment development, has traditionally been focus of fierce debate [15]. When developing his attachment theory, Bowlby was in a difficult position. On the one hand he wanted to emphasise that attachment is an evolutionarily primed behavior system, not reducible to classical or operant conditioning [16]. On the other hand, he also held that the attachment behavioral system is assembled and elaborated in the context of learned experiences [17]. Nevertheless, he mostly left the topic alone. Later, attachment researchers mostly demonstrated that children develop their attachment relationships independent of the quality of care, interpreting this finding as evidence that the attachment behavioral system is an evolutionary driven system that requires no learning experiences to be established [15, 18].

More recently, researchers started to argue that this biological preparedness of infants to establish attachment relationships with caregivers does not fully explain why individual differences in children's expectations or trust about caregivers' availability for support develop [8]. One thusfar understudied possibility could be that learning models might be a helpful addition to attachment theory to explain these differences [19]. Recent literature argues in a similar way that adult attachment development reflects a conditioning process [20-22]. Focusing on childhood attachment, Bosmans [23] proposed that children learn to trust (CR) in a responsive primary caregiver like the mother (CS) when the mother gets associated with the repeated experience that she provides successful support during distress (UCS), which is automatically followed by a sense of relief and a sense of security (UCR). At the level of operant conditioning, it has been proposed that trust versus lack of trust in support during distress (Sd) increases or decreases the likelihood that children will seek support (R) during distress. More versus less support seeking is respectively reinforced by the fact that sensitive caregivers help to solve problems more easily or by the fact children avoid experiencing the anticipated negative effects of rejection or inadequate support [23].

*In addition, we referred back to this discussion in the discussion section:*

p29: Thus, it would be premature to argue that the current study contradicts attachment theory's traditional view that attachment is an inborn mechanism that does not require learning processes [18]. A next step would be to investigate whehter these findings translate to more naturalistic research. With that regard, it is promising that the current pattern of findings does not contradict existing more broadband attachment research.

**3. There are times when the discussion (which appears frequently in the results section) seems to get too far from the data and the limitations of the approach. The following two sentences are examples, but not the only instances:**

*Overall, we tried to be more careful in phrasing our interpretations. With regard to the specific examples, we made the following changes:*

**"So, this was the first evidence suggesting that good enough mothering requires at least 80% contingent care”.**

We now wrote:

"So, if this finding could be replicated in more naturalistic research on parent-child interactions, the finding could be used as an argument that good enough mothering might require at least 80% contingent care."

**"These results suggest that negative care-related experiences outweigh prior positive learning experiences.”**

*We now wrote:*

"This again seems to suggest that trust catches up after exposure to more positive

experiences, but that more negative prior learning experiences persist reducing trust levels."

**Similarly, the authors found that trust in one's caregiver was related to baseline trust in a "novel caregiver", though the correlations were low (r = .24 and r = .16) with only one truly significant. These data are not overwhelmingly convincing - I think if we posited the hypothesis that trust within these two contexts was different, we'd find ourselves with some evidence to suggest that they are. Could the authors provide a more constrained reporting of the meaning of these results?**

We now wrote:

"However, in favor of the paradigm’s relevance was the finding that baseline trust in the novel supportive figure was slightly but significantly correlated to trust in the mother in Study 3. Nevertheless, we need to stay cautious interpreting this correlation, as it could not be fully replicated in Study 2. This might have been due to reduced power, but could also indicate that we created a less relevant experimental context. This all suggests that more research and similar research with more ecologically valid designs is crucial in the future,"

**4. The authors start the paper seeking to justify their experimental approach. While I understand their desire to point out the limitations of previous research, I wonder if they may go too far with sentences like “Clinically, broadband correlational studies are difficult to translate into concrete therapeutic strategies.” I’m not sure that they are, and there are treatments that have been developed based on this research. The authors may want to find another way to introduce their study, particularly because the major limitation of the current study is that ecological validity is decreased by the experimental approach with “new caregivers”, which may make it quite difficult to translate these findings into the clinical realm.**

*We agree with this concern, and when rereading the paper, we felt that the setup of the study did not really require such a clinical argument. Consequently, we decided to drop this sentence from the introduction.*
