# Supplementary material for: Trust development as an expectancy-learning process: Testing contingency effects
Source: PLoS One. 2019 Dec 11;14(12):e0225934. doi: 10.1371/journal.pone.0225934 (PMC6905544; doi:10.1371/journal.pone.0225934)
Supplement: Attachment — Submitted filename: Responses to the reviewers_Final.docx [file pone.0225934.s005.docx]

**Additional Editor Comments:**


***Please also carefully review the percentage of contingencies through the sections on Study 3. There seem to be inaccuracies in 50 vs. 70 vs. 100 compared to the test contingencies of 20 vs. 80. vs. 100.***

We are grateful that you noticed this error and changed this in the results section of Study 3.

**Reviewer #1:**

***Overall I think this is a very important contribution to the attachment literature. Attachment theory needs to be integrated with learning theory. Whereas Bowlby created a compelling overall model of attachment and how it develops, given the paucity of data on neurobiology at the time it inadequately addresses the fundamental mechanisms underlying attachment learning. Although one may argue that the underlying mechanism of attachment is an innate behavioral system, and therefore no standard learning mechanisms can explain attachment dynamics, I believe this is a fundamental error in both reading Bowlby and for the future of attachment theory writ large. Bowlby argues for a goal corrected behavioral system. This implies learning. The mechanisms he uses to explain such learning are based in a perspective of homeostasis that no longer comports with our understanding of physiological regulation. It is clear that learning processes are subserved by general purpose neural systems that function in a manner well characterized by learning theory, and that those systems are critical for the regulation of physiological and psychological needs. Bowlby was working in a time when behaviorists were too narrow minded about the breadth of basic rewards and punishments. They completely ignored the possibility that social contact was a very real need in the same vein as food and water. Moreover, their blank slate view of all animals was unsustainable. As such theorists like Bowlby filled that vacuum with models that did not rely on learning theory based mechanisms. Unfortunately for the field it has taken far too long to reconcile and integrate Bowlby's critical contributions with those of the behaviorists and cognitive learning theorists that followed. This is an important step in that direction and I applaud the authors for their creativity and boldness in furthering this integration.***

We were very grateful for these words of praise, as they express exactly what we hoped to achieve through this study.

**Reviewer #2:**

***The authors have made some major revisions to their manuscript which I think improve the overall product. At the same time, I still think their conclusions go well beyond the bounds of their research design in places and could be further qualified. I have specific feedback below:***

We are happy that the reviewer deems our changes in line with the majority of his/her prior requests. We agree that the additional suggestions indeed were necessary to allow readers to more adequately evaluate the contribution of the studies to the literature.

***Point 2. The authors make a valiant effort to discuss and resolve the tension between the role of learning theory in attachment and attachment as an innate biological system. I personally think there is a distinction between the processes of the attachment system, which seem to be innate, and attachment styles that are likely (at least partly) shaped by learning. It may be worthwhile to differentiate their discussion in this way, as they have started in their added text, to some degree. Attachment theorists often propose a model akin to learning theory to explain the development of anxious, avoidant or secure attachment styles, while at the same time noting Bowlby’s regard for attachment processes (meaning proximity seeking, secure base, etc.) as primary needs. Researchers often discuss, for instance, that secondary attachment styles are still ways to maintain attachment (e.g., proximity to caregiver) in non-ideal contexts (e.g., parent is rejecting of attachment needs). Delineating what is/can be learned may be more innate is an important discussion for this paper.***

We are grateful that the reviewer was in agreement with the main points of the adjustment we made in response to the prior request. We do agree that the added suggestions are in line with the message that we wanted to communicate, so we adjusted these parts of the introduction so they would be more explicit in making the point that above an innate system the development of different attachment styles might reflect at least partly learning processes. We adjusted this part as follows (we underlined the changes we made):

"More recently, researchers started to argue that this biological preparedness of infants to establish attachment relationships with caregivers does not fully explain why individual differences in children's expectations or trust about caregivers' availability for support develop [8]. One thus far understudied possibility could be that learning models might be a helpful addition to attachment theory to explain attachment-related differences that are not innate [19], such as secondary (anxious, avoidant, and secure) attachment styles (Mikulincer & Shaver, 2017). Recent literature argues in a similar way that adult attachment development reflects a conditioning process [20-22]. Focusing on childhood attachment, Bosmans [23] proposed that children learn to trust (CR) in a responsive primary caregiver like the mother (CS) when the mother gets associated with the repeated experience that she provides successful support during distress (UCS), which is automatically followed by a sense of relief and a sense of security (UCR). At the level of operant conditioning, it has been proposed that trust versus lack of trust in support during distress (Sd) increases or decreases the likelihood that children will seek support (R) during distress. More versus less support seeking is respectively reinforced by the fact that sensitive caregivers help to solve problems more easily or by the fact children avoid experiencing the anticipated negative effects of rejection or inadequate support [23]. In sum, the current study builds on the idea that the processes of the attachment system are innate, but that attachment styles are (at least partly) shaped by learning."

***3. I still feel like these phrases are too much for the research method to support. There may quite a bit different about interacting with a real attachment figure during a critical timepoint in life that makes a lower percentage of contingent care necessary, for instance. I would advice deleting this claim, and all others like it. The authors show a proof of concept, which is interesting, but I would not try to push for the idea that we are really seeing attachment-like processes unfolding. The data don't seem to support tat either (see 4).***

We agree that we could be even more cautious in positioning the studies within the broader area of attachment research and in interpreting the findings. Therefore, from the introduction onwards, we introduce the current studies as testing a proof of concept explaining why we opted to use this paradigm (focusing on a novel attachment figure and specifically on help provided by this figure). In the results sections, we avoided using language that suggests that we can extend our interpretations beyond what we found with this paradigm. Finally, in the discussion, we more carefully point again at the fact that this is a proof of concept study and that real-life attachment interactions might show different dynamics. Now we only make the point that the findings suggest that it could be useful to study contingency-related dynamics in attachment-relevant interactions with real attachment figures.

In the introduction, we now wrote:

(p6): "The current study aimed to provide proof of concept for the idea that contingency could explain part of the variance in the development of trust (Research Question 1) and in the development of support seeking (Research Question 2) and for the idea that questions regarding the definition of good enough mothering and regarding the dynamics of (in)stability of trust over time can in theory be explained by contingency-related effects (Research Question 3)."

(p7): " Although this approach will not straightforwardly translate to the complex interactions with a real attachment figure during critical timepoints in individuals' lives, the approach has the advantage that we can identify the role of contingency controlling for the impact of prior learning experiences with those real attachment figures. This way, we can experimentally test whether differences in contingency affect trust and test how changes in contingency from the learning to the test phase are linked to changes in trust. Finally, we can measure participants’ inclination to seek the caregiver’s help during the test phase, as a proxy of participants’ support seeking behavior."

In the results section of Study 1 we now wrote:

(p. 13): "This suggests in adult participants that initial contingency effects had a lasting influence in trust development, even after exposure to a novel, improved experience."

In the results section of Study 2 we now wrote:

(p. 16): " Like in Study 1, this suggests that trust development depended on the contingency of success in provided care and that trust development in the current paradigm required more than 70% contingent caregivers."

(p. 19): " Like in Study 1, this suggests that improving contingency increased trust, but that prior less contingent care experiences delayed this catch-up."

In the introduction to study 3 we removed the sentence that we were looking for an indication of how much contingency is needed to receive good enough mother. The sentences now are written as follows:

(p. 20): " Moreover, Study 2 did not reveal which percentage of contingency suffices to increase trust levels compared to baseline. Therefore, we included an 80% instead of a 70% contingent condition, to test whether 80% contingency suffices to significantly increase trust."

In the results section of Study 3 we now wrote:

(p. 21): " So, this suggests for the current experimental procedure that contingency needs to be at least 80% to be good enough for trust in the novel caregiver to increase."

(p.24): " This again seems to suggest that trust caught up after exposure to more positive experiences, but that more negative prior learning experiences persistently reduced trust levels."

(p.24): " These results suggest that negative care-related experiences in our experiment outweighed prior positive learning experiences."

In the general discussion, we now wrote:

(p. 26) " Results were largely in line with the predictions, providing first proof of concept that trust development might be studied from a conditioning perspective. Moreover, at a more detailed level, results suggested that more experimental research on the effects of contingency on trust development could provide new insights in the developmental dynamics underlying trust-related fluctuations, and eventually regarding the development of attachment."

(p. 26): " This finding is in line with the existing broadband research showing that more sensitive and responsive parenting is linked with elevated levels of trust in parental support [5], but adds to this research because it provides proof of concept that contingency could at least partly explain the causal mechanism underlying this link and because it suggests that it might be important to account for the relative contribution of single learning experiences across trust development. "

(p 28): " The current proof of concept results suggest that studying contingency effects on trust development might be especially interesting to investigate the effect of changes in the quality of care over time. So far, such research could only be conducted in so-called naturalistic experiments like adoption [39]. However, adoption remains rare and the impact of adoption is hard to distinguish from other factors related to child characteristics, pre-adoption experiences, and post-adoption experiences. Like in adoption research, the current findings obtained with our paradigm do suggest substantial catch-up in trust development and support seeking if adverse learning circumstances are followed by more beneficial circumstances [40]. The results also suggest that the initial learning effects continued to have an effect even after a more positive learning experience. This raises the hypothesis that prior more negative experiences have a lasting negative impact on trust. Instead, more positive learning experiences seemed quite sensitive to negative changes in the quality of the caregiver's response. Interestingly, these changes seemed to have had a stronger effect at the level of expectations than at the level of behavior. Real life attachment research could test whether, indeed, children are inclined to continue seeking support in spite of a drop of trust. This illustrates how our hypothesis and paradigms like the one we used in the current studies to yield proof of concept could lead to novel developmental hypotheses that can be tested more directly with more naturalistic research paradigms. "

(p. 29-30): " In spite of these limitations, the current findings are theoretically relevant. In three studies, we found evidence that trust development, and related support-seeking behavior, might be understood from learning theories regarding classical and operant conditioning. More specifically, results suggest that trust might develop as an expectancy-learning process with contingency (likelihood that help is successful) as a fundamental mechanism explaining differences in trust and support seeking. This finding is in line with the increasing awareness that trust development underlies a cognitive learning process resulting in the development of a secure base script [9]. This insight could help improve theory about trust development and could help shape interventions aimed at restoring trust in order to remedy the development of emotional and behavioral problems. Through this approach, we might be able to identify for individual children the level of contingency they need to increase their trust in the availability of their caregiver. With such information, it becomes possible to design interventions that promote trust development. For example, we could start creating the circumstances in which contingency of the caregiver is more guaranteed."

***4. The authors now note that their lack of a significant finding of a correlation between trust in a “novel caregiver” and trust in the mother may be due to reduced power. However, the effect size is simply extremely small in the case of study 2. Even if this were significant, given the author’s claims that they are measuring attachment processes is problematic when there is such a small relationship between these measures. I would omit the mention of reduced power being the fault and more clearly state that these data may argue against the same process being measured in both instances.***

In line with this request, we omitted the discussion of power, emphasized more the small effect sizes we found, and added the suggested message that interpretation of the current studies' results require caution.

(p. 27): " However, we need to stay cautious interpreting this correlation, because the effect size in Study 3 was small and because the effect could not be fully replicated in Study 2. So, it is not unlikely that we have not measured exactly the same process as it occurs in real-life attachment relationships. Nevertheless, the robustness of the majority of our findings suggests that more research and similar research with more ecologically valid designs might be critical to further advance attachment theory. "
